# Antiparasitic Activity of Mirazid, Myrrh Total Oil and Nitazoxanide Compared to Praziquantel on *Schistosoma mansoni*: Scanning Electron Microscopic Study

**Published:** 2017

**Authors:** Mona EL-SAYAD, Sahar ABU HELW, Hend EL-TAWEEL, Mohammad AZIZ

**Affiliations:** Dept. of Parasitology, Medical Research Institute, Alexandria University, Alexandria, Egypt

**Keywords:** Mirazid, Mice, Myrrh, Nitazoxanide, Praziquantel, Scanning electron microscopy, *Schistosoma mansoni*

## Abstract

**Background:**

The development of new antischistosomal drug remains a pressing need and a vital challenge in front of many researchers through screening the natural or chemical substances for their potential activity as antischistosomal agents.

**Methods:**

Five groups of *Schistosoma mansoni*-infected mice (n=10) were enrolled in this study, the G1 was infected non-treated control group. G2 was infected treated with praziquantel 500 mg/kg for 2 consecutive days. G3 was given mirazid 500 mg/kg for 5 days. G4 was given Myrrh total oil 18 mg/kg for 3 days and G5 given nitazoxanide 100 mg/kg for 7 days. Mice perfusion was performed for worm ultrastructural morphology by scanning electron microscopy at 2 WPT.

**Results:**

Praziquantel was superior to any other tested substances as it caused extensive tegumental damages in male worms in the form of rupture of the tubercles and loss of spines followed by mirazid that resulted only in superficial tegumental damage with shrinkage of the outer surface of male tubercles with marked loss of spines. Nitazoxanide resulted in minor tegumental alterations of male worms while Myrrh total oil caused negligible effects on the teguments of perfused worms.

**Conclusion:**

PZQ showed more superior antiparasitic effects than all tested substances on *S. mansoni* worms. Mirazid was more effective than myrrh total oil and nitazoxanide.

## Introduction

The tegument of *Schistosoma* has been described as a living, anucleate, and cytoplasmic structure covering the outer surface of the worm (1). This tegument has a role in the synthesis and secretion of various nutrients, absorption of nutrients and shields the worms from the immune response by the infected host (2, 3). Scanning electron microscopy (SEM) has become a useful tool for the study of the ultrastructural alterations on the surface of the schistosomes in response to chemotherapy by showing the effect on the tegumental structures (tubercles, spines, and inter-tubercular ridges), oral and ventral suckers (4). The ultrastructural changes in the tegument of *Schistosoma* worms are directly proportional with the antischistosomal potency of these drugs (5) and may clarify the procedure of killing these worms (6). Focal damage in the tegument induced by an antischistosomal drug might be repaired over the course of 7–14 days effectively after cessation of the drug while in severe tegumental damage; the host immune response might affect (7). Increased exposure of *Schistosoma* antigens (epitopes) at the parasite surface accompany these morphological changes (8) leading to the disappearance of the immunological ’disguise’ of the worm and inability to engulf food by oral and ventral suckers. This is believed to be of prime importance in causing death of the worms (9, 10).

Treatment of this parasitic disease provides a double benefit: it reduces both the morbidity caused by the adult worms in the human host and the excreted eggs to the environment. Treatment depends on only one drug all-over the world, praziquantel (PZQ) that is pyrazinoisoquinoline derivative initially synthesized as potent tranquilizer. Then, its anthelmintic activity was tested and proved at the laboratories firstly as a veterinary drug for cestodiasis. Subsequently, it was the only available drug active against all *Schistosoma* in vitro and in vivo animal and human clinical trials (11). PZQ is the first broad spectrum anthelmintic pharmaceutical product to fulfill the WHO’s requirements for population-based chemotherapy (12). It is advisable to look for alternate drugs to fight this disease (13). Considerable efforts have been made to develop novel antischistosomal drugs either by synthesis of new chemical entities or preparation of extracts or compounds from the natural resources or by drug repositioning of the already available drugs to save time and costs of preclinical toxicological evaluation and phase 1 clinical studies (14–17).

Mirazid (MZD) had been a new natural preparation applied to be logged in the Egyptian market since 2002 (18) as a new treatment of schistosomiasis to avoid the hazards achieved by the extensive use of PZQ as an only drug for more than 3 decades as well as it is effective in all patient categories in adults and children. In addition, it can be used in all cases of schistosomiasis complicated with hepatosplenomegaly (19). In spite of many promising results achieved in clinical trials of MZD for the treatment of schistosomiasis (20), the drug was unable to be marketed internationally in endemic countries due to the influence of the passive impact of many other experts reported its ineffectiveness and even recommended not to use it in control programs (21). The founder of the myrrh oleoresin combination known as MZD proved that this combination is more effective in schistosomiasis treatment than the myrrh volatile oil hence marketed the combination on the expense of the oil (22). However, a recent finding reported that the volatile oil is more effective than the oleo-resin combination and concluded that to gain much benefits either by increasing the oil percent in MZD or lodge the oil only on the expense of the combination that is already available and marketed as MZD (23) and new formula was developed using only the volatile oil of myrrh (24).

Nitazoxanide (NTZ) is a nitrothiazol derivative structurally related to the anthelminthic and molluscicidal agent, niclosamide (16). It was FDA-approved as an antiprotozoal drug for the treatment of diarrhea caused by *Cryptosporidium* and *Giardia* in children in Dec 2002 and in adults in Jul 2004 (25). Data about the antischistosomal action of NTZ are not clear. Administration of NTZ (orally with 100 mg/kg once or twice daily for 4 d at 42 d post-infection with 140 cercariae by S/C injection) in a murine model of schistosomiasis *mansoni* decreased hepatic egg count by 34% and significantly improved hepatic and spleen pathology although no effect on worm burden could be observed (16).

This study aimed to assess the antiparasitic activity of MZD and its derivative **(**Myrrh Total Oil, MTO) as well as NTZ compared to the reference drug, PZQ in *S. mansoni*-infected mice by using the scanning electron microscopic examination of the perfused worms 2 wk post-treatment (WPT).

## Materials and Methods

### Materials

**1-Experimental animals:** The study included 50 Eight-wk-old female Swiss albino mice (*Mus musculus)* of the CD-1 strain.

**2-Parasite strain:** Laboratory-bred *Biomophalaria alexandrina* snails infected with miracidiae of Egyptian (CD) strain of *S. mansoni* were obtained from the *Schistosoma* Biologic Supply Center (SBSC), Theodore Bilharz Research Institute (TBRI), Cairo; Egypt.

**3-The drugs:** PZQ was obtained as biltricide tablets from Alexandria Company for pharmaceutical and chemical industries, Batch No: 9118014. MZD capsules were obtained as free medical samples from Pharco Pharmaceuticals, Batch No: 296. Myrrh total oil was obtained from Safe Pharmaceuticals (Safe-pharma). Nitazoxanide was obtained from as nitazode powder for oral suspension from Sigma Pharmaceuticals, Batch No: 21581.

**4-**Instruments used in scanning electron microscopic study (Incubator, Critical point dryer, Fine coater, Scanning electron microscope and Computer) from the electron microscopy unit, faculty of science, Alexandria University, Alexandria, Egypt.

### Methods

**1-Mice infection with S. *mansoni* cercariae:** Infected *B. alexandrina* snails were washed with dechlorinated tap water and kept in an aquarium in an aerated (by using electric pump) in a dark place under white fluorescent light for a period of 30–60 min to release cercariae. Number of cercariae was counted and the average number per 1 ml was calculated according to the method (26). Mice were infected using paddling technique (27) with 100 cercariae/mouse. Stool examination was performed 45 d after cercarial infection to investigate the presence of *S. mansoni* eggs (28).

**2-Study Design:** Fifty Swiss albino mice were used in this study and were divided into 5 equal groups of 10 mice each.

Group1: Infected non-treated control group given 0.2 ml of the drug vehicle (cremphor EL).

Group 2: Infected and treated with the standard drug, PZQ in a dose of 500 mg/kg for 2 d (29) which is the therapeutic dose in mice based on Food and Drug Administration guidelines for converting the human dose to those for experimental animals but it should be given once.

Group 3: Infected and treated with MZD at a dose of 500 mg/kg for 5 days. The measurement was chosen as indicated (30, 31) fourfold the therapeutic dose in mice (125 mg/kg) and it should be given for 6 d as recommended by the manufacturer.

Group 4: Infected and treated orally with MTO 18 mg /kg /day for 3 d (22, 32).

Group 5: Infected and treated orally with NTZ 100 mg/kg for 7 consecutive d (16) with some modifications.

**3-Scanning Electron Microscopic Study**: Mice were sacrificed and perfused to collect the worms from each group at 2 wk post-treatment. Male worms were washed in saline and then fixed in a fixative mixture containing 2.5% glutaraldehyde and formaldehyde in 0.15 M phosphate buffer (pH 7.2) at 40C. Dehydration was carried out at room temperature through an ascending graded acetone series (30%–100%) and followed by critical point drying using liquid carbon dioxide (Samdri-PVT-3B, Tousimis, USA). Specimens were mounted on aluminum stubs with double-sided adhesive carbon and then coated with gold (fine coater). The specimens were examined in the Electron Microscope Unit, Faculty of Science; University of Alexandria by using SEM model (Jeol-JSM-5300) according to Bricker et al. (33).

The study protocol was reviewed and approved by the Ethics Committee of the Medical Research Institute (MRI), University of Alexandria.

“All applicable international, national, and/or institutional guidelines for the care and use of animals were followed.” “All procedures performed in studies involving animals were in accordance with the ethical standards of the institution or practice at which the studies were conducted.”

## Results

**Fig. 1:**
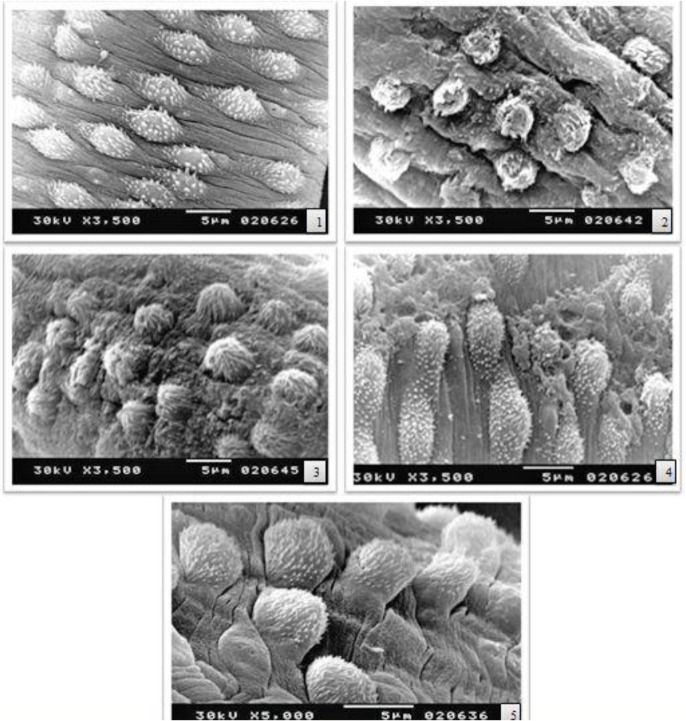
Scanning electron micrographs of the tegument of male *S.mansoni* worms recovered from different groups (1) infected non-treated, (2) PZQ, (3) MZD, (4) NTZ, (5) MTO.

PZQ caused a pronounced tegumental damage with rupture of the tubercles and loss of spines in wide areas in male worms exposing the underlying muscle layers (Fig.1). MZD caused considerable tegumental damages with shrinkage of the outer surface. Besides, it resulted in marked loss of spines of male worms if present, loss of their sharpness. NTZ resulted in mild tegumental damaging effects manifested by focal lesions in the inter-tubercular ridges without effect on tubercles and spines. MTO showed no antiparasitic activity, as it caused no alteration in the tegument of male worms (Fig.1).

## Discussion

Treatment of *S. mansoni* infected mice seven WPI with 500 mg/kg for 2 d by PZQ resulted in extensive damages in the tegument of the recovered worms with rupture of the tubercles and loss of spines in wide areas exposing the underlying muscle layers. These results were in accordance with several in vitro and in vivo studies on *Schistosoma* worms under the effect of PZQ (8, 9, 29, 33–43).Treatment with MZD 500 mg/kg in murine model of *S. mansoni* infection showed considerable antiparasitic effects on the perfused worms at 2 WPT manifested by tegumental damages with shrinkage of the outer surface. It also resulted in marked loss of spines of male worms if present, loss of their sharpness. These results agreed with some reports (6, 41, 44), but did not agree with the other results (43) who reported that the dorsal and ventral surfaces of *S. haematobium*– recovered from MZD-treated hamster (500 mg/kg for 3 d) were intact 3 months after treatment. The myrrh total oil showed minute changes in the tegument of male worms recovered after treatment of infected mice with 18 mg/kg for 3 days. The low efficacy of MTO noticed in this work may be explained by a difference in the nature of the used oil (22), as it was examined the myrrh volatile oil prepared by hydrodistillation. However, in this study, the total oil of myrrh was prepared by solvent extraction with petroleum ether percolation method as directed (32) as there is a difference in the chemical components and consequently the potency between the two oils (45). NTZ resulted in mild effects on the recovered worms from infected mice treated with 100 mg/kg for 7 d manifested by focal lesions in the inter-tubercular ridges without effect on tubercles and spines. These results were contradictory to the findings that reported no effect of the drug on the schistosomes (16).

## Conclusion

PZQ showed more superior antiparasitic effects on *S. mansoni* worms than all tested substances. MZD was more effective than MTO and NTZ.
